# Bayesian Network Analysis of Intervention-Induced Physical Activity Behavior Change: Comparative Modeling Study Across Age, Education, and Activity Impairment Subgroups

**DOI:** 10.2196/57977

**Published:** 2025-09-03

**Authors:** Simone Catharina Maria Wilhelmina Tummers, Arjen Hommersom, Lilian Lechner, Roger Bemelmans, Catherine Adriana Wilhelmina Bolman

**Affiliations:** 1 Department of Health Psychology, Faculty of Psychology, Open University of the Netherlands, Valkenburgerweg 177, Heerlen, 6419 AT, The Netherlands, 31 455762888; 2 Institute for Computing and Information Sciences, Radboud University, Nijmegen, The Netherlands; 3 Department of Computer Science, Faculty of Science, Open University of the Netherlands, Heerlen, The Netherlands; 4 Lectorate Data Intelligence, Zuyd University of Applied Sciences, Heerlen, The Netherlands

**Keywords:** physical activity, physical exercise, subpopulation differences, behavior change, age, education, physical activity impairment, Bayesian network, eHealth, e-health intervention, digital health, digital technology, digital intervention, multiple studies, demographics

## Abstract

**Background:**

Tailoring intervention content, such as those designed to improve physical activity (PA) behavior, can enhance effectiveness. Previous Bayesian network research showed that it might be relevant to tailor PA interventions based on demographic factors such as gender, revealing differences in determinants’ roles between subpopulations. In order to optimize tailoring, one needs to understand the differences between subpopulations based on different characteristics. Building on this, this study examines age, education level, and PA impairment as key moderators, as these factors might influence PA engagement and intervention responsiveness. Older adults, for example, rely more on habitual behavior, lower-educated individuals may face challenges due to lower health literacy and socioeconomic inequalities, and individuals with PA impairment, defined as functional impairments restricting PA, may face unique barriers to PA. Understanding differences based on these factors is crucial for optimizing interventions and ensuring effectiveness across diverse populations.

**Objective:**

This study investigates, by means of Bayesian networks, differences in PA intervention mechanisms of subpopulations based on age, education level, and PA impairment.

**Methods:**

Subpopulation-specific subsets from an integrated dataset of 5 studies are analyzed, including demographics, experimental group assignment, and PA and sociocognitive measures at baseline, short term, and long term. The relevant subpopulations are defined based on age, education level, and PA impairment. For each subpopulation, a stable Bayesian network is estimated based on the corresponding subset of data by applying a bootstrap procedure and according to a confidence threshold, relevant paths of the model are visualized in order to find indications regarding subpopulation-specific intervention mechanisms.

**Results:**

A comparison of subpopulation-specific models unveils similarities and differences with respect to determinants’ roles in PA behavior change induced by interventions. Similar structures of determinants affect short-term PA, ultimately causing effects in the long term, where intention and habit are directly related to PA for most subpopulations. With respect to age-based differences, the interventions influence PA less via attitude cons and planning for older than younger people. Looking at the level of education, planning and intrinsic motivation are less influential for low-educated participants compared with high- or medium-educated participants, whereas more influence takes place through attitude pros for this low-educated group with respect to maintaining effects in the long term. Looking at PA impairments, apart from the findings that attitude pros and planning are more relevant in the pathway of change for people without impairment, a more interesting insight is that fewer determinants are directly influenced by the intervention within the group with PA impairment.

**Conclusions:**

Intervention mechanisms in specific demographic groups have been rarely studied so far. Initial interpretations from the derived subpopulation models in this study unveil subpopulation-specific patterns of behavioral change, which enable better tailoring of intervention content to characteristics of the target population in order to induce or enhance effects.

## Introduction

Despite known health benefits of physical activity (PA), adherence to the PA guidelines of the World Health Organization is low across different subpopulations [1-7]. E-health interventions have been designed to improve PA behavior of different target subpopulations, for example, Dutch-speaking adults older than 50 years [8], independently living, Dutch-speaking adults older than 64 years experiencing PA impairment [9], or (former) adult patients with prostate and colorectal cancer [10]. Such behavioral change intervention programs are designed based on behavioral change theories such as the theory of planned behavior and self-determination theory [11,12]. Based on these theories, behavior change strategies are incorporated into the programs in order to influence psycho-social determinants of PA behavior [13,14]. Determinants are factors expected to be directly or indirectly correlated with PA behavior, and it is presumed that changes in behavior are reached by influencing these determinants [8,9,13,15-18]. Tailoring of interventions based on an individual participant’s characteristics, resulting in personalized intervention content, has proven effective [19-21]. It is known that different subgroups, such as vulnerable people with low socioeconomic status (SES), are at an increased risk of physical inactivity and respond in diverse ways to specific parts of a PA intervention [22]. For example, previous research highlights the importance of considering significant barriers such as fear of injury and promoting awareness of the benefits of PA in designing PA interventions for older adults [23]. Comparable, it is known that for people with and without PA impairment [9,22], the determinants of PA are different, something that is also likely for people with different levels of education [24]. Understanding the concrete differences between subpopulations with regard to PA behavior change mechanisms would allow tailoring to be optimized in order to improve intervention effects and the sustainability of these effects. Results of a recent study by Tummers et al [25] already give rise to tailored interventions for gender-specific subpopulations. This emphasized the importance of conducting further research into possible other factors defining subpopulations that are relevant for tailoring.

Previous research [25] has revealed knowledge about concrete roles of key determinants in PA behavior change, which is relevant information for designing and tailoring future interventions. In this study, an overview of complex interactions between a PA intervention, determinants, and PA outcome levels was provided for a general population of older adults (50 years and older) and gender-specific subpopulations [25]. The importance and roles of determinants in short-term (6 mo after the baseline) PA behavior change and maintenance of intervention effects in the long term (12 mo after the baseline) have been shown for these groups. In general, habit and intention appeared to be directly related to PA outcome levels. Social concepts, self-efficacy, attitude, intrinsic motivation, commitment, and planning all play a relevant indirect role, affecting PA via other determinants. The models demonstrate that most determinants influence short-term PA, and that these effects permeate into the longer term via previous PA or the direct determinants attitude pros (ie, perceived advantages of PA) and intention. Regarding the differences between males and females, the role of self-efficacy differs across both subpopulations, and attitude is shown to be more relevant for males, while planning turned out to be more important for females. These insights into the working mechanisms of PA interventions have been gained by analyzing an integrated dataset of multiple intervention studies applying a machine learning approach based on Bayesian networks [22,26-30]. Since this methodology provided valuable insights, especially with regard to subpopulation-specific differences, it is important to study the relevance and role of determinants of PA of other subpopulations besides gender, similarly. Potentially relevant moderating factors are age, education level, and PA impairment, as explained below.

Age has been identified in several studies as a moderator of PA behavior change [22,24,31]. In addition, the type of PA behavior has been shown to depend on age [32]; domestic activities make up a greater proportion of total moderate- to vigorous-intensity PA among older adults in contrast to exercise, fitness, and team sport activities. Therefore, differences in age groups are studied. Further, vulnerable groups are often related to low health literacy, making it relevant to see to what extent people’s degree of education or their SES affects the PA behavior change mechanisms [33]. Despite mixed findings and varying measurement approaches complicating interpretations and explanations, a review study showed that there is an association between SES and PA [34], where a higher SES is generally associated with greater PA in adolescents. In addition, educational level has been shown to be a moderating factor of intervention effects on PA, examined by analyses for subgroups, where different levels of short-term (household-activity related) PA increases were found for education-based groups of intervention participants [22,28]. Finally, it is important to identify differences in PA behavior change between people with and without PA impairment, as determined by the presence of functional impairments that hinder PA [27]. People experiencing PA restrictions are shown to be less physically active than those without physical impairments [27]. Furthermore, experiencing limitations in physical activities makes it much more difficult for individuals to increase their PA; previous research has shown that personal barriers such as an impairment should be considered when advising on sports and that the presence of physical impairments is a moderator of intervention effects on PA [22,35]. Therefore, it is relevant to look into the specific mechanism for people with any restriction in physical functioning compared with those without PA impairment.

Previous literature has thus shown differences between subpopulations defined by the factors age, education, and PA impairment; however, little insight remains in how these factors relate to the roles of determinants. Because of that, it is expected that it is relevant to further examine these subpopulations. Therefore, in this paper, we explore differences in PA intervention working mechanisms of the subpopulations based on age, level of education, and being physically limited. This study uses a Bayesian network analysis for each of these subgroups in order to identify differences and similarities in the relevance of determinants and their roles in the short- and long-term per moderating factor. Since the factors studied are shown to be relevant moderators, it is expected to find differences that can provide useful information for optimizing tailoring interventions based on these factors.

## Methods

### Case Description

This study analyzes a compound dataset of 5 different studies on the effectiveness of PA interventions, previously developed and evaluated, that focus on changing PA behavior through computer-tailored personal advice, selected based on the similarity in the design of the intervention content [22,25,27-30,36]. For completeness, information about the effectiveness and main characteristics of the included studies with respect to intervention design is presented, leading to differences in emphasis of content (Table 1). To get an overall overview, beyond personalized intervention content, the data from multiple studies have been integrated based on study-overarching concept definitions. This study investigates general differences in intervention effects and mechanisms across the defined subpopulations. Thereto, models are estimated for subpopulation-specific subsets of the dataset, defined by a moderating factor.

**Table 1.** Characteristics and effectiveness of included intervention studies [25].

Study index (reference)	Intervention target group	Intervention delivery mode	Effectiveness at 6 and 12 months after baseline
1 [18]	Adults aged 50 years and older	Written	Yes. Depends on intervention content (more specifically, only the intervention including environmental information is effective) and differs between age-, BMI-, and intention-specific subgroups.
2 [8]	Adults aged 50 years and older	Written and digital per individual	Yes. Depends on delivery mode and environmental conditions (no intervention effect found in web-based environmental conditions). Also, for some conditions, the moderation effects of age, gender, and intention have been found.
3 [16]	Single, adults aged 65 years and older	Written and digital	No. Effects at 3 months, but these have evaporated at 6 months. No measurement at 12 months.
4 [9]	Adults aged 65 years and older having chronic or physical limitations for PA^a^	Written and digital	Yes, although limited. Depends on the degree of impairment, BMI, age, and educational level.
5 [17]	Former/current patients with cancer aged 18 years and older	Written and digital	Yes. Depends on cancer type, educational level, gender, and age.

a PA: physical activity.

In this study, relevant subpopulations based on the factors age, level of education, and PA impairment were defined. A fairly recent study shows that many older adults (older than 65 years) have no intentions to be physically active [37]. Differences in intention and also in lifestyle (eg, working vs retired) led to the decision to distinguish between the subpopulations of participants younger than 65 years and participants being 65 years or older. In order to gain insight into mechanisms for vulnerable groups with low SES, this study focuses on the behavior change of less educated individuals compared with average or higher-educated people. Thereto, the classification for education level defined by the Dutch governmental institution Statistics Netherlands [38] is adhered to in this study. Further, participants who reported having PA impairments are compared with participants who were not physically limited. This study adopts a broad classification of individuals as either being limited in their PA or not, in order to account for variations in underlying chronic conditions and in the degree of physical limitations across individuals and studies. This approach aligns with the focus of the interventions on promoting PA rather than on specific medical conditions. The heterogeneity within the group with PA impairments should be acknowledged, as individuals within this group may have different perceptions towards PA and encompass various underlying conditions. Despite this, they share the common challenge of facing additional physical barriers to perform PA compared with the other, healthier group, making tailored intervention strategies particularly relevant.

### Study Data

As mentioned, an integrated dataset derived from data of the 5 e-health intervention studies is the subject of this study [22,27-30,36]. Effects in such intervention studies are measured by the collection of data from intervention participants as well as participants in a (waiting-list) control group, who have not received any intervention content during the experiment (Table 1 describes the effects). The control group of each study is comparable to the experimental group of the corresponding study, and participants of the control group received intervention content after the experiment phase and final measurements. All participants received questionnaires at different moments in time, namely at the baseline (T0, just before receiving intervention content), and at 3 (T1), 6 (T2, short-term effects), and, in most studies, 12 (T3, long-term effects) months after the baseline [8,9,16-18]. Due to differences in measured variables and measurement timing across studies, missing values emerged during dataset integration. In this paper, analyses are performed on subpopulations, that is, subsets of the integrated dataset including the most important variables that were also analyzed in previous, related research [25]. For each subpopulation for which a Bayesian network is estimated, a relevant subset of records is taken from the integrated dataset, depending on participants’ values for the factor that is analyzed. For validation, the numbers of control and intervention group participants for each subpopulation that is analyzed are presented (Table 2).

**Table 2.** Number of participants in the control and intervention conditions for each investigated subpopulation group. Due to missing values in the demographic factor variable, the total number of participants (in the control or intervention group) of the 2 subgroups for the different evaluated factors may not match.

Subpopulation	Number of participants	
	Control group	Intervention group
Age (years)		
<65	643	2019
≥65	922	2126
Education		
Low	682	1597
Medium to high	734	2153
Physical activity impairment		
Without	666	2186
With	790	1777

### Measurements

The integrated dataset consists of PA outcomes, the main socio-cognitive determinants of PA, demographics, and the intervention variable (Table 3).

The outcome measure of total minutes of moderate- to vigorous-intensity PA per week is central to the analyses in this study. This outcome measure of intervention effects on PA behavior is derived from the raw data of the self-administered Dutch Short Questionnaire to Assess Health-Enhancing PA (SQUASH) included in the questionnaires that participants received [39].

**Figure 1.**
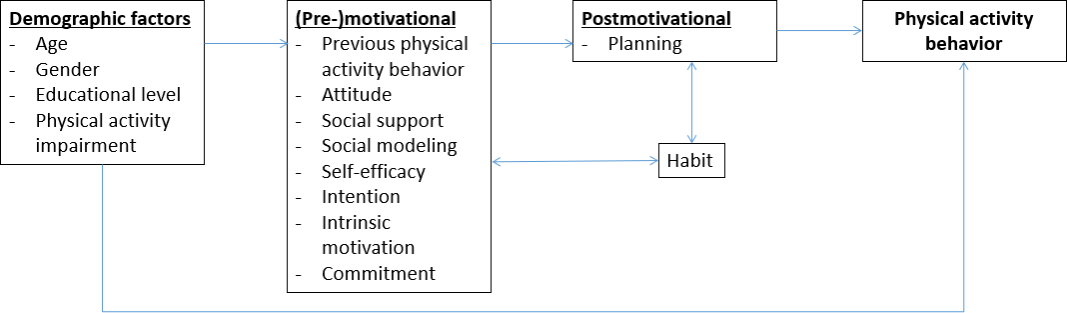
Theoretical intervention framework for the role of determinants in influencing physical activity [40-42].

The core determinants are selected based on the studies included in the analysis, requiring that each determinant be measured in at least 2 studies. The underlying intervention studies draw on theory- and evidence-based models, which demonstrated the relevance of these determinants to PA behavior. The selected determinants are self-efficacy, attitude (pros and cons), intrinsic motivation, intention, commitment, strategic planning, action planning, coping planning, habit, social modeling, and social support. The premotivational determinants of self-efficacy refer to an individual’s expectation about their own capacity to engage in PA, and attitude reflects an individual’s positive and negative evaluation of PA. With respect to the motivational determinants, intrinsic motivation refers to the extent to which an individual engages in PA for personal enjoyment or inherent satisfaction, that is, without external influence, and intention to the extent to which an individual plans or aims to engage in PA. The postmotivational determinant commitment concerns the extent to which an individual is dedicated to maintaining PA over time. With respect to the different (postmotivational) planning determinants, strategic planning involves formulating an explicit approach engaging in PA, action planning refers to specifying when and how to perform PA, and coping planning entails adjusting plans to remain active despite unexpected circumstances. The final postmotivational determinant habit refers to the extent to which PA is performed automatically and consistently as part of an individual’s routine. Finally, determinants related to an individual’s social environment are included, namely social modeling referring to the influence of observing the behavior of others , where individuals may adopt PA patterns based on examples in their social environment, and social support reflecting the direct encouragement or assistance an individual receives from others, such as family or friends, to engage in PA. Figure 1 shows information about determinant roles from established behavior change models [40-42]. The definitions described above are also derived from these models. As depicted, the determinants interact at different stages of PA behavior change, where premotivational determinants influence an individual’s awareness and readiness to engage in PA, motivational determinants drive the decision to initiate PA, and postmotivational determinants support the maintenance of PA over time by facilitating consistency and overcoming barriers [43]. The determinant concepts mentioned were assessed by several items, and corresponding concept variables are aggregated from the raw item data. Note that the operationalization of concepts differs to some extent across the included studies. All measures were self-reported. The concept scales are computed as the mean of the corresponding unipolar item scales, allowing a maximum of 25% missing items per concept. Except for intention, which was measured on a 10-point scale, items were measured on a 5-point scale, and the number of items per concept varies across the studies and across concepts.

**Table 3.** Timeslots of measurement of determinants and outcomes (including indication of studies) [25]. For the subpopulation younger than 65 years, no data are available from studies 3 and 4, and for the subpopulation without impairments, limited data are available from study 4.

Variable/timeslot	Baseline (T0)	3 months (T1)	6 months (T2)	12 months (T3)
Self-efficacy	X (1,2,3,4,5)^a^	X (1,2,3,5)	X (5)	X^b^ (4)
Attitude (pros and cons)^c^	X (1,2,3,4,5)	X (1,2,3,5)	X (5)	X (4)
Intrinsic motivation	X (1,2,5)	X (1,2)	X (5)	—^d^
Intention	X (1,2,3,4,5)	X (1,2,3)	X (1,2,4,5)	X (1,2,4,5)
Commitment	X (1,2)	X (1,2)	X (1,2)	—
Strategic planning	X (1,2,3,4,5)	X (1,2)	X (1,2,5)	X (1,4)
Action planning	X (1,2,5)	X (1,2)	X (1,2,5)	—
Coping planning	X (1,2,5)	X (1,2)	X (1,2,5)	—
Habit	X (1,2,4,5)	—	X (1,2,4,5)	X (1,2,4,5)
Social modeling	X (1,2,3,4,5)	X (1,2,3)	X (1,3)	X (4)
Social support	X (1,2,3,4,5)	X (1,2,3,5)	X (3,5)	X (4)
SQUASH^e^ outcome (MVPA)^f^	X (1,2,3,4,5)	X (1,2,3,5)	X (1,2,3,4,5)	X (1,2,4,5)

a "X" indicates that a variable was measured at a specific time point. The numbers in parentheses refer to the studies in which it was measured at that time point.

b Raw data measurements are very limited.

c Attitude pros and attitude cons have been measured and are included in analyses as separate concepts.

d The concept was not measured at that specific time point.

e SQUASH: Short Questionnaire to Assess Health-Enhancing Physical Activity.

f MVPA: moderate- to vigorous-intensity physical activity.

Each of the sociodemographic factors, age, gender, educational level, and PA impairment, is either included in a particular model, or the focus of the analysis if the investigated subpopulations are defined based on the demographic. In analyses where the subpopulation under consideration is not defined based on age, this demographic is uncategorized and measured in years. Gender and PA impairment are included as Boolean variables. Educational level is categorized into low, medium, and high, according to the Dutch education system [38]. In analyses in which this demographic defines the subpopulation under consideration, the medium and high-educated subgroups are merged into a single category.

A Boolean intervention variable is included in analyses in this study, indicating whether a participant was part of the control or the intervention group. It should be noted that the content that intervention participants received is unique for each individual because of computer-tailored advice, as mentioned in the background section. However, in tailoring, the intervention content is altered minimally, based on the answers of respondents to a large number of tailoring questions. Therefore, the personalized intervention content is beyond the scope of this paper but can be found in previous papers [8,9,16-18].

### Analysis

In this study, a Bayesian network approach is applied to evaluate differences in PA behavior change mechanisms between subpopulations defined by the demographic factors age, educational level, and PA impairment, respectively. The experiments were conducted in R (version 4.0.3; The R Foundation for Statistical Computing), and the package bnlearn (version 4.6.1) was used for Bayesian network learning [44,45]. Additionally, the package igraph (version 1.2.6) was used to extract desired paths from the Bayesian network models, mpmi (version 0.43.1) for the estimation of mutual information, and Rgraphviz (version 2.34.0) to visualize the Bayesian networks [46-48]. Different models are learned from a subset of the data for which one of the moderating factors has certain values of interest, and this (constant) factor has been left out of the model. By taking a subset of the data, it is possible that for some variables, there is no data at all and has thus been excluded from the analyses of a specific subpopulation. Each model considers a subpopulation defined by one of the demographic factors mentioned and corrected for the other 2 demographic factors and gender.

Bayesian networks are probabilistic models that represent relations between variables based on conditional independencies and that can be represented as directed acyclic graphs [49]. The Bayesian network models that are learned are temporal and hybrid, assuming a multinomial and Gaussian distribution for the discrete and continuous random variables, respectively. The networks are restricted to ensure that arcs are consistent with the time dimension of measurements.

To estimate a Bayesian network that includes a stable set of arcs, the same approach as in previous, related articles is used [25], applying Efron’s Bootstrap [50]. To explain this approach briefly, we chose the number of bootstrap samples ranging from 100 to 150 based on the structural Hamming distance stability measurement, for a chosen confidence threshold. To learn a Bayesian network model for each bootstrap sample, an algorithm is applied that combines structure learning with the estimation of missing values, called the structural expectation maximization algorithm [51]. The model structure is learned by searching through candidate graphs and selecting the structure that best fits the data according to the Bayesian Information model selection Criterion [52,53], under the time dimension-related restrictions. An averaged model is created based on the bootstrap sample models, in which arcs are selected with at least 60% confidence. It should be noted that, in case both directions occur equally often in the bootstrap sample models, relations in the averaged model are considered undirected.

**Figure 2.**
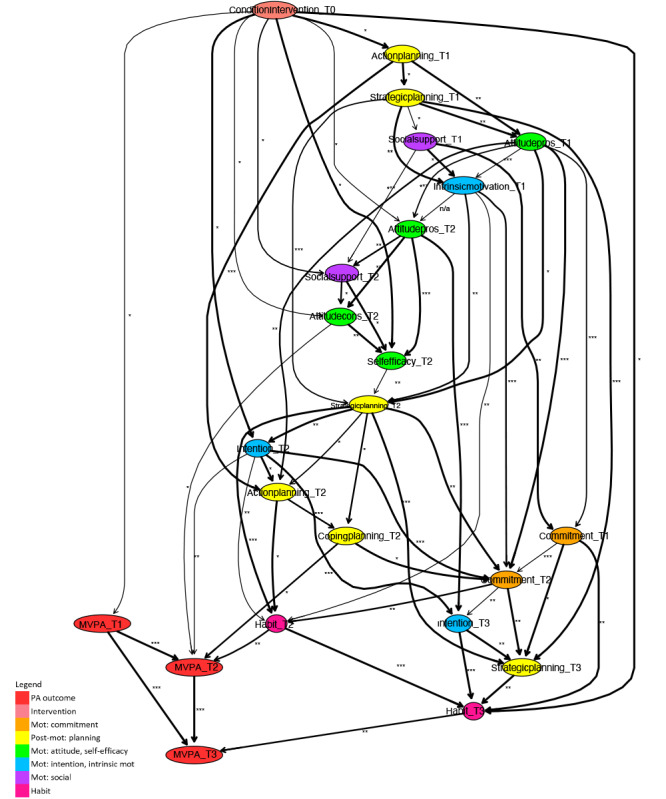
Fragment of a Bayesian network learned for a subpopulation of participants younger than 65 years. Mot: motivation; MVPA: moderate- to vigorous-intensity physical activity; PA: physical activity.

**Figure 3.**
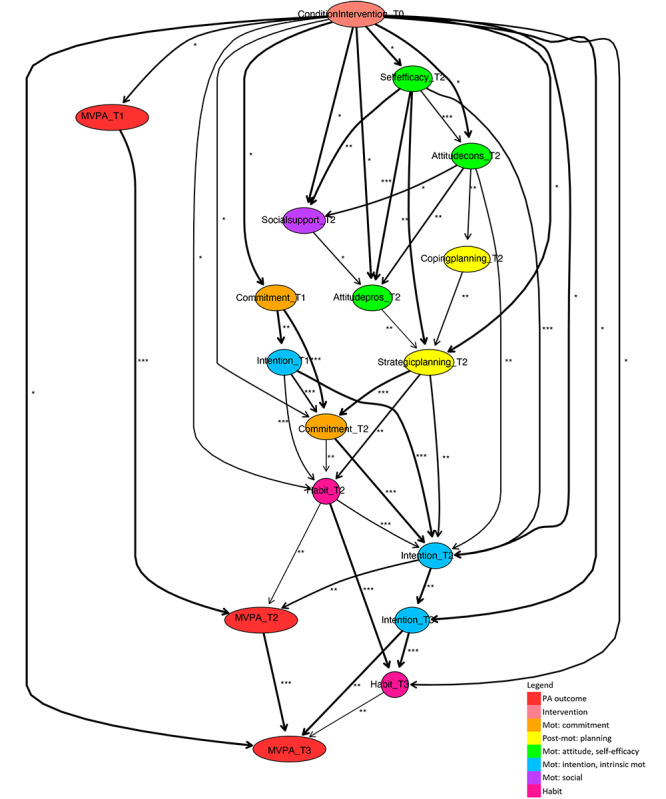
Fragment of a Bayesian network learned for a subpopulation of participants aged 65 years and older. Mot: motivation; MVPA: moderate- to vigorous-intensity physical activity; PA: physical activity.

**Figure 4.**
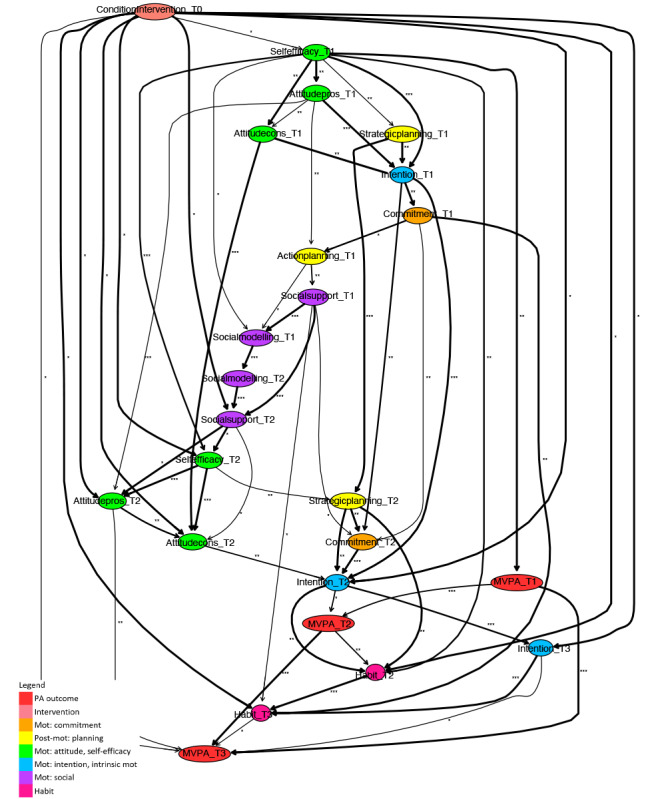
Fragment of a Bayesian network learned for a low-educated subpopulation. Mot: motivation; MVPA: moderate- to vigorous-intensity physical activity; PA: physical activity.

**Figure 5.**
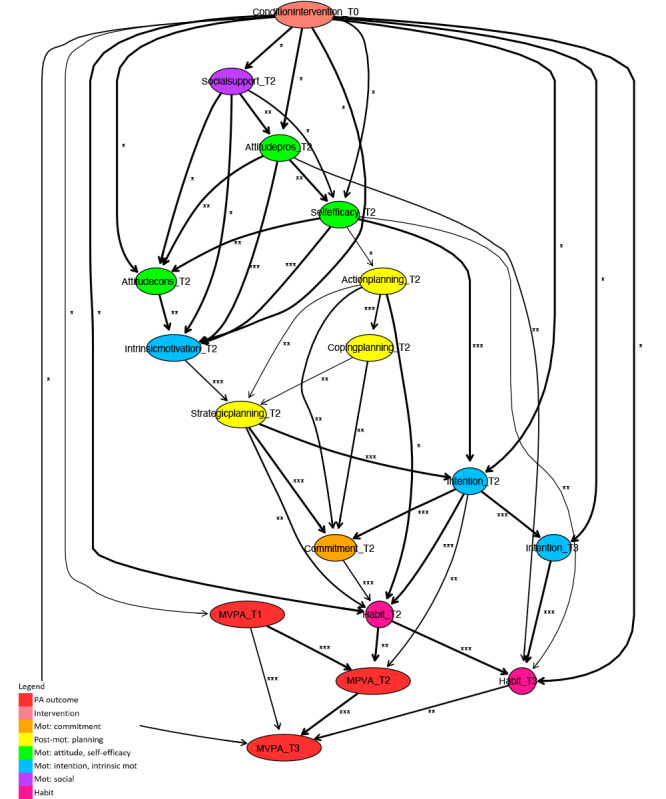
Fragment of a Bayesian network learned for medium- and high-educated subpopulation. Mot: motivation; MVPA: moderate- to vigorous-intensity physical activity; PA: physical activity.

**Figure 6.**
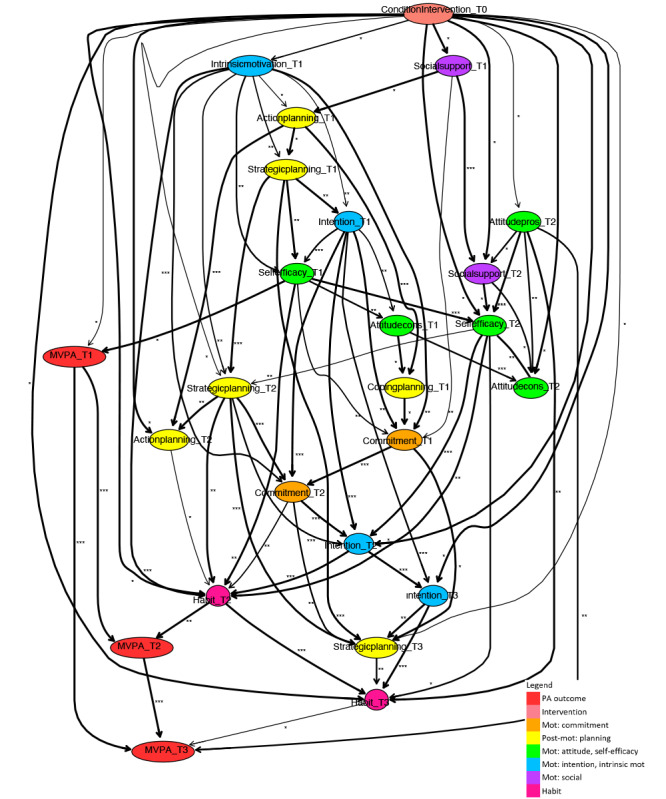
Fragment of a Bayesian network learned for a subpopulation without physical activity impairment. Mot: motivation; MVPA: moderate- to vigorous-intensity physical activity; PA: physical activity.

**Figure 7.**
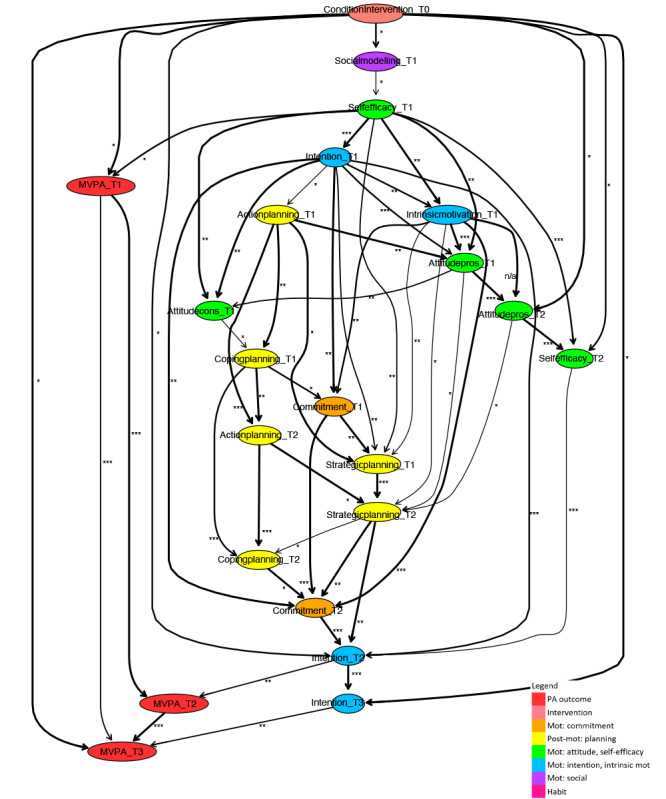
Fragment of a Bayesian network learned for a subpopulation with physical activity impairment. Mot: motivation; MVPA: moderate- to vigorous-intensity physical activity; PA: physical activity.

To draw conclusions from the derived averaged model regarding PA intervention working mechanisms, the relevant paths between the intervention condition variable and the short- and long-term PA outcome variables are deduced. For interpretation purposes, the model is visualized, where the strength of each relation is indicated by asterisks and the stability in the arcs’ thickness (Figures 2-7). The asterisks are derived from the jack-knife bias-corrected mutual information estimates, based on complete cases, and relatively allocate arcs to 3 groups by cutting off at 33% and 67% quantiles [54]. Note that the exact cut-off points are given in the results section for each model presented. The different arcs’ thickness levels divide arcs according to the percentage of bootstrap sample models in which the specific arc occurs, as described, being at least 0.6, into 4 groups, cutting off at 0.7, 0.8, and 0.9. To simplify the comparison of final models with respect to relevant determinants and their roles in pathways of intervention influences, different colors of nodes indicate determinant categories.

### Ethical Considerations

The 5 studies in this analysis’s dataset [22,25,27-30,36] received ethics approval from the research ethics committee (Commissie Ethische Toetsing Onderzoek) of the Open University of the Netherlands. Institutional review board approval numbers can be found in the original publications. All participants in the original studies provided informed consent. No additional ethics approval was required for the present analysis, as it involves secondary analysis of fully anonymized pre-existing data. According to Dutch national regulations, such research falls outside the scope of the Medical Research Involving Human Subjects Act (WMO) [55]. Furthermore, institutional guidance from the Open University of the Netherlands’ research ethics committee (Commissie Ethische Toetsing Onderzoek) confirms that no ethical assessment is required for studies using fully anonymized secondary data [56]. The authors of the previous studies made the corresponding data available and gave permission for secondary data use.

## Results

### Overview

This section presents the distilled paths of the averaged Bayesian networks learned from the PA intervention data for subpopulations defined by age, educational level, and PA impairment of participants. First, differences between participants younger than 65 years and 65 years and older are considered. Second, groups of lower and medium-to-high-educated participants are evaluated. Third, PA intervention working mechanisms of participants with PA impairment compared with those without are examined. Thereby, the number of bootstrap samples to learn a model was determined per subpopulation based on the structural Hamming distance (Multimedia Appendix 1). Due to the complexity of the resulting models, the focus of this study is limited to the main similarities and differences that are most profound in comparing the 2 models under consideration for age, education, and physical impairment subgroups, respectively. Since most direct influences of the intervention on determinants are relatively weak, the main effect pathways were selected in the presented submodels based on their stability. Note that influences induced by these weakly affected determinants are relatively stronger, ultimately effectively leading to behavioral change. For the declaration of findings, overviews of the means and SDs of variables in the subpopulation models are available (Multimedia Appendices 2-4).

### Age

#### Overview

Segments of the Bayesian network were learned, similarly following the procedure described in the analysis section, for data of only participants younger than 65 years (Figure 2) and participants aged 65 years and older (Figure 3). The following variables have been removed from the age-based subpopulation sets, since there were no observations for the subpopulation younger than 65 years: self-efficacy at T3, attitude pros at T3, attitude cons at T3, social modeling at T3, and social support at T3. It should be noted that the total integrated dataset consists predominantly of data for older adults, aged 50 years and older.

#### Short-Term Age-Related Paths

Considering the paths of short-term PA intervention behavior change, for both subpopulations, habit and intention at T2 have a direct relation with short-term (T2, after 6 mo) PA outcomes. In addition, the pathways of intervention effects on these determinants are similar. Indirect determinants that play an important role in these paths are attitude cons, attitude pros, self-efficacy, social support, strategic planning, and commitment at T2.

There are also important differences between the 2 subpopulations. First, attitude cons, and coping planning at T2 have a direct relation with short-term PA outcomes for the subpopulation younger than 65 years, while for the subpopulation aged 65 years and older, they have an indirect influence. Second, for the subpopulation younger than 65 years, action planning at T2 is related to short-term PA indirectly, via habit at T2, while this determinant does not appear in the model for the subpopulation aged 65 years and older. Third, because the direct influence of the intervention on action planning at T1 is highlighted (ie, distilled) in the model for the subpopulation younger than 65 years, several other determinants at T1 appear in this model, while they do not appear in the model for the subpopulation aged 65 years and older. For the younger subpopulation, these determinants at T1 ultimately create effects on the pathways that have been discussed to be similar for both subpopulations, where attitude pros at T1 seem important to predict the sequel.

#### Long-Term Age-Related Paths

For both subpopulations, most of the intervention effects in the long term originate from short-term pathways. Habit and intention at T3 are ultimately influenced by these pathways and play an important role in long-term effects, where habit at T3 is a direct determinant of PA at T3.

The role of intention at T3 differs between subpopulations. For the subpopulation aged 65 years and older, this is a direct determinant of PA at T3, while for the subpopulation younger than 65 years, the influence of this determinant is indirect and causes effects on PA at T3 via habit at T3. Another difference between the 2 subpopulations is that for the subpopulation younger than 65 years, strategic planning T3 occurs in the long-term pathways, while it does not occur for the subpopulation aged 65 years and older.

### Education

#### Short-Term Education-Related Paths

For both subpopulations (Figure 4, where the edge between attitude cons and intention at T1 is undirected, and 5), habit and intention at T2 are interrelated and both directly related to short-term PA outcomes. These direct determinants are influenced by the intervention directly and indirectly. Within the structure of intervention effects on these determinants, in the models for both subpopulations, the determinants social support, attitude (pros and cons), self-efficacy, commitment, and planning at T2 have similar (indirect) roles.

There are some differences between the 2 subpopulations regarding the occurrence of determinants, as well as the arc direction. First, intrinsic motivation at T2 does not occur in the intervention pathways for lower-educated people, but does occur in those for medium- to high-educated people, where it has an indirect role in the PA intervention behavior change mechanism due to its effect on strategic planning. Second, there is a difference between the 2 subpopulations with regard to which specific planning determinants play a role. At T2, in intervention paths for lower-educated people, only strategic planning is relevant, while for medium- to high-educated people, action planning and coping planning are also important. Third, the direction of the arc between habit and PA at T2 is different, which can be due to the fact that some structures that differ in arc direction model the same probability distribution, that is, they can be Markov equivalent. Fourth, it should be noted that at first glance, the models suggest large differences between the 2 subpopulations, though in reality, differences may be smaller. Namely, several determinants at T1 occur in the model for lower educated, which ultimately have effects on the corresponding structure discussed earlier (which broadly implies that the intervention influences social and attitudinal determinants, which, potentially through planning and commitment, affect the direct determinants of PA at T2, namely intention and habit) and do not appear in the model for medium- to high-educated. Because the relationship between the intervention and self-efficacy T1 is highlighted, a whole chain of determinants at T1 is also shown in the model. Indeed, it could be the case that this single relation of the intervention and self-efficacy at T1 was just not stable enough for the subpopulation of medium- to high-educated, so that a similar chain of relationship probably present in the whole model has not been highlighted in the submodel.

#### Long-Term Education-Related Paths

For both educational subpopulations, the models show how long-term intervention effects (ie, at T3) arise from short-term effects, especially through previous PA and because of the permeation of effects on habit and intention into the longer term. Another similarity is that habit at T3 is a direct determinant of PA at T3.

An important long-term difference is that attitude pros at T2 and intention at T3 have a direct influence on PA at T3 for lower-educated people, while for medium- to high-educated people, these determinants have only an indirect influence (via habit at T3).

### Physical Activity Impairment

#### Short-Term Paths Related to Physical Activity Impairment

Although there is a difference in short-term direct determinants (ie, at T2) of the 2 subpopulations (Figure 6, where the edge between self-efficacy and attitude cons at T2 is undirected, and 7), the pathways through which intervention influences arise on these key determinants are roughly similar. Within this structure, self-efficacy and attitude influence planning and commitment, which in turn influence habit or intention. In both models, determinants at T1 also occur. Intrinsic motivation and intention at T1 play an important role because of their direct relation with many of the determinants within the discussed structure.

As already briefly mentioned, there is a difference in direct determinants within PA intervention pathways between the 2 subpopulations. Intention at T2 is a direct determinant of short-term PA for participants with PA impairment, while it has an indirect influence for participants without PA impairment. For participants without PA impairment, habit at T2 is a direct determinant of short-term PA, while this determinant does not occur in the model for participants with PA impairment. Another difference is in direct intervention effects on determinants. For participants without PA impairment, most determinants are directly influenced by the intervention, whereas for participants with PA impairment, only some are directly influenced.

#### Long-Term Paths Related to Physical Activity Impairment

For both subpopulations, long-term intervention effects arise from short-term pathways, but the role that the determinants play in it mainly differs. The direct determinants for one subpopulation have a more indirect, or even no role, for the other subpopulation. For participants with a PA impairment, intention at T3 is the direct determinant of PA at T3, while it has a more indirect role for participants without PA impairment. For participants without PA impairment, the direct determinants of long-term PA are habit at T3 and attitude pros at T2. For participants with PA impairment, habit at T3 does not occur, and attitude pros at T2 only has an indirect role because of its role in the short-term pathways of intervention effects. Beyond this, strategic planning at T3 has an indirect relation via habit at T3 with long-term PA for participants without PA impairment, while it does not occur at T3 for participants with PA impairment.

## Discussion

### Principal Findings

Overall, the structure of short-term pathways of intervention effects corresponds to the compared subpopulations, and long-term effects arise from those pathways. Habit and intention are important determinants of short-term PA, as for most subpopulations in the intervention pathways, they have a direct relationship with PA at T2. Only for participants without PA impairment does intention have an indirect role, and for participants with PA impairment, habit is not important. In addition, attitude cons and action planning play a more important role in the pathways for the population younger than 65 years than those for 65 years and older. Further, intrinsic motivation and planning variables play a less important role for the low-educated than for the medium- to high-educated. In summary, determinants play an important role in the short-term pathways of intervention effects for one subpopulation, while they have none or a more indirect role for the opposing subpopulation. With regard to direct intervention influences, there is a direct relation between the intervention and many determinants in most subpopulation models considered, which means that the intervention is capable of changing determinants. However, for the subpopulation of people with PA impairment, few determinants are directly influenced by the intervention. For example, intrinsic motivation, planning concepts, and habit are directly influenced by the intervention for the subpopulation without PA impairment, but not for participants with PA impairment. The upcoming section elaborates on the implications associated with insights from this study.

### Methodological and Interpretation Issues

To increase statistical power, which is particularly important in case analyses that focus on subdatasets, data from multiple studies are integrated. Partly stemming from the data integration process, analyses in this study had to deal with substantial amounts of missing data. In dealing with this, a method for dealing with missing data that has been evaluated within a similar context in previous research was applied [57]. Through a bootstrap procedure, we have generated robust outcomes and gained insights into the stability of relations in the models. Directions of arcs in the resulting models were determined based on bootstrap sample models and temporal restrictions. Few undirected edges were observed in the resulting models, which means that there is uncertainty about the exact direction of certain relations. While this complicates the interpretation of results, it reflects the complexity of a network of interactions in which multiple variables are interconnected.

With a focus on the study objectives, paths have been distilled from the models that also meet a stability threshold, which was determined while considering false positives and false negatives of the identified relationships. These paths provide a comprehensive and reliable overview of the mechanisms of subpopulations. In particular, this study focuses on vulnerable groups such as people with low education, who are known to have low health literacy. Previously, there was limited insight into intervention mechanisms for specific target groups. However, network models are complex in nature, posing a challenge in comparing them and extracting key accent differences. One can potentially derive substantial information from the subpopulation models about how to adapt and tailor interventions in order to enhance their effects. An initial step has been taken in interpreting these models in the context of adaptation. Further research on the impact of adjustments on intervention effects should reveal the optimal approach for translating mechanism models into effective modifications.

Note that different determinants at T1 appear in the model for those younger than 65 years and in the model for the low educated, which do not appear in other subpopulation models. Because of the highlighted fragments from models (ie, not all arcs/edges are visualized), one has to be careful not to get an inaccurate idea about the differences between subpopulations. More specifically, the occurrence or nonoccurrence of a whole pathway of determinants in a fragment, for example, may depend on the decision whether the influence of the intervention on just one of those determinants is stable enough to be highlighted. In view of these and other results, it is worth keeping in mind during interpretation that the models shown represent specific paths and the most stable relations, as part of a larger model. Furthermore, it is important to note that the results in this study have solely compared subpopulations defined based on a single demographic factor. To gain a more in-depth understanding of how to approach tailoring optimally, it might be necessary for future research to examine subpopulations defined based on combinations of demographic factors as well.

### Comparison of Results With Prior Work and Their Practical Implications

#### Overview

In this section, it is explored how the research outcomes from this study relate to existing knowledge in the context of PA behavior change. Note that whereas previous research has zoomed in a little specifically on the working mechanisms for different demographic groups, this study focused on subgroup-specific mechanisms. Based on the described findings about these specific PA intervention mechanisms, indications can be provided on how intervention contents can be tailored to induce and enhance long-term effects for the investigated subgroups. Several key points are highlighted based on the main differences. Note that further accent variations could be derived from the models presented in this study.

#### Comparison to the General Population

In earlier, related research on PA behavior change induced by interventions for a general population of (mainly) older intervention participants, a similar behavior change structure was found for short-term PA, with the same direct determinants (ie, habit and intention) and long-term effects that follow from this [25]. Beyond these similarities between subpopulations, which are also consistent with findings in a general population, in this paper, important differences are presented for subpopulations defined by age, educational level, and PA impairment. These differences imply that some determinants are more relevant in the paths of intervention effects for certain subpopulations or have a slightly different role in these paths. As with the difference between males and females previously studied [25], the insights into subpopulation-specific differences provide valuable information about how to tailor interventions.

#### Subpopulations Defined by Age

Previous research emphasizes differentiated approaches in intervention development, highlighting age as a factor that should be considered in tailoring interventions for specific target populations [58,59]. This study has revealed some nuanced differences regarding determinants that play a slightly more prominent role for certain age groups, such as attitude cons and action planning. Furthermore, prior research has indicated that specific groups exhibit distinct motives for PA, such as health aspects for older participants and social aspects for younger participants [58]. The scientific knowledge about differences in motives is valuable in the context of PA determinants. Motives underlie many of the determinants. There are, for example, particular motives that contribute to the creation of PA habit for a specific subgroup. Findings from this study suggest that determinants such as attitude cons and action planning play a more important role in pathways of intervention effects for people younger than 65 years compared with those for people aged 65 years and older. These findings can be explained by the greater flexibility of younger adults to translate intentions into behavior, whereas older adults, who may already have higher motivation, are less likely to rely on motivational or cognitive factors and more likely to rely on habitual behavior [60,61]. For older adults, information on creating and maintaining habits may be particularly beneficial. To tailor interventions age-based and promote PA behavior change in the younger population, it might be more effective to emphasize these determinants. Note that insights about underlying motives are useful in determining how to affect the designated determinants.

#### Subpopulations Defined by Education

Previous research recommends developing tailored strategies to promote PA among socioeconomically disadvantaged groups, acknowledging the challenges these groups face in sustaining PA [62]. This study provides suggestions on how this can be achieved. For instance, different roles are found for the planning concepts, indicating that intervention elements that aim to stimulate action and coping planning approaches might be less effective for low-educated people, while improving strategic planning techniques may be as beneficial for low as for medium- to high-educated individuals. These findings can be explained by differences in cognitive processing and self-regulation skills across educational levels [63,64]. Lower-educated individuals may be less accustomed to structured planning techniques, while strategic planning approaches focusing on habit formation appear to be beneficial regardless of education level. In addition, concerning the maintenance of effects in the long term, intention and attitude pros play a more crucial role for low-educated individuals compared with their medium- to high-educated counterparts. Lower-educated individuals may rely more on intentions and attitudes for behavior change due to limited access to resources and less developed self-regulation skills, making their personal motivation more central in determining their behavior. To tailor interventions based on education, focusing on PA of lower-educated people, strategies aiming to enhance the determinants of strategic planning, intention, and attitude pros may be more impactful to enhance PA. Just as with age groups, previous research has provided insights into specific motives as well as barriers related to PA for various social groups [65]. In the implementation of strategies aimed at influencing key determinants identified in this research for the low SES group, one might consider their particular motives and barriers. For example, if financial position is an important barrier, strategies should address affordable alternatives.

#### Subpopulations Defined by Physical Activity Impairment

Also, for the factor of experiencing restrictions in physical functioning, some research has been conducted on the motives and barriers of relevant subgroups [66]. Previous research has also provided limited insights into determinants of PA for the subgroup of individuals with PA impairment [67], namely that intrinsic motivation and self-efficacy are important. This research confirms this, but also indicates that these determinants play similar roles for individuals without PA impairment. Internal drivers thus facilitate overcoming obstacles to PA. Further, it has been found that habit does not play a role in intervention paths for the group of individuals experiencing PA impairments, probably because PA is more difficult due to experienced impairments and might therefore not be as ingrained in their daily routines, making it less likely to develop into an automatic behavior. More importantly, results in this study show that the intrinsic motivation of participants with PA limitations is not directly influenced by the intervention and that, in general, fewer determinants are directly influenced for the subpopulation with PA impairments. This may indicate that the differences between subgroups based on the moderating factor of the presence of a PA impairment are the greatest, and that advice may not fit the situation of participants with reported physical limitations. In relation to this, a more detailed examination of the degree of PA impairment would be valuable for future research. To tailor interventions in order to enhance the effectiveness of interventions for the subpopulation with PA impairment, it may be beneficial to implement strategies to affect apparently less responsive factors, such as intrinsic motivation and habit, that address their specific needs and challenges related to PA. The effect for this group may be strengthened by, in the intervention design, taking into account intrinsic barriers and key motives such as physical improvement and rehabilitation [66].

### Conclusions

This study has focused on understanding the working mechanisms of PA interventions for specific subgroups. It has been shown that PA interventions induce very complex change processes, with the interventions influencing PA behavior through different pathways, both direct and indirect. For the different subgroups, based on age, education, and PA impairment, similarities but also relevant differences in the pathways of behavioral change are found. While further research is certainly important, current insights can be used to make interventions more effective by suiting them even better to the important characteristics of the target population, in particular, vulnerable groups. This study contributes to improved health and well-being within these subpopulations and to reducing health inequalities.

## Supplementary material

10.2196/57977 Multimedia Appendix 1 Stabilization of averaged Bayesian network subpopulation models during the bootstrap procedure.

10.2196/57977 Multimedia Appendix 2 Overview of the mean (SD) of variables appeared in age-based subpopulation models. Because the response rate differs across groups and analyses are based on available data, the n value may differ for the different analyses

10.2196/57977 Multimedia Appendix 3 Overview of the mean (SD) of variables appeared in education-based subpopulation models. Because the response rate differs across groups and analyses are based on available data, the n value may differ for the different analyses.

10.2196/57977 Multimedia Appendix 4 Overview of the mean (SD) of variables appeared in physical activity impairment-based subpopulation models. Because the response rate differs across groups and analyses are based on available data, the n value may differ for the different analyses.

## Abbreviations

PA: physical activity
SQUASH: Short Questionnaire to Assess Health-Enhancing Physical Activity
