# Supplementary figures and images for: Bayesian Network Analysis of Intervention-Induced Physical Activity Behavior Change: Comparative Modeling Study Across Age, Education, and Activity Impairment Subgroups
Source: Online J Public Health Inform. 2025 Sep 3;17:e57977. doi: 10.2196/57977 (PMC12407225; doi:10.2196/57977)

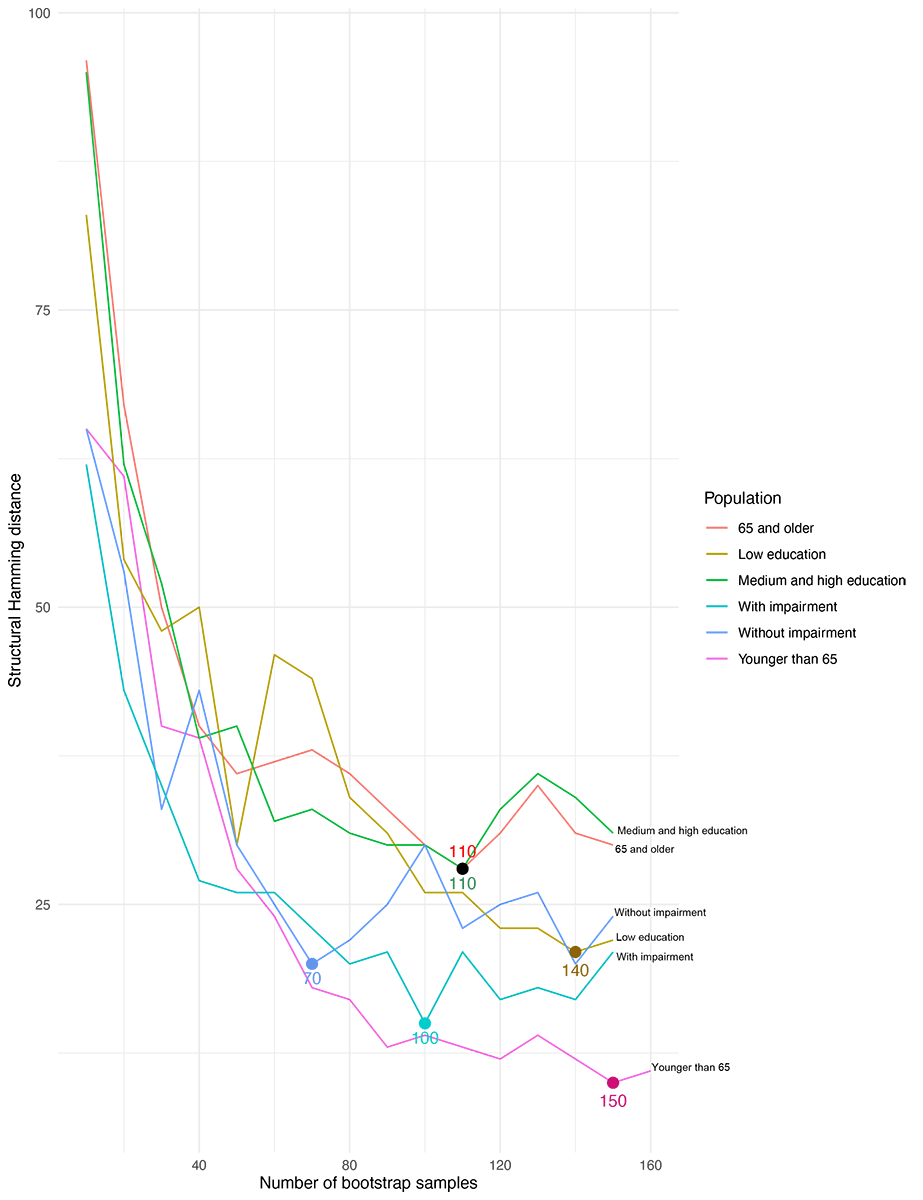

Supplement: Multimedia Appendix 1 [file ojphi-v17-e57977-s001.png]
