# Supplementary material for: Bayesian Network Analysis of Intervention-Induced Physical Activity Behavior Change: Comparative Modeling Study Across Age, Education, and Activity Impairment Subgroups
Source: Online J Public Health Inform. 2025 Sep 3;17:e57977. doi: 10.2196/57977 (PMC12407225; doi:10.2196/57977)
Supplement: Multimedia Appendix 2 [file ojphi-v17-e57977-s002.docx]

| **Determinant (timeslot) / Subpopulation** | **Younger than 65** | | **65 and older** | |
| --- | --- | --- | --- | --- |
|  | **Control** | **Intervention** | **Control** | **Intervention** |
| PA (T1) | 677.88 (668.94) | 807.19 (683.12) | 888.86 (748.65) | 967.53 (844.98) |
| PA (T2) | 721.92 (727.12) | 892.73 (806.20) | 827.90 (737.00) | 911.18 (763.51) |
| PA (T3) | 624.71 (598.15) | 758.47 (728.64) | 854.45 (827.81) | 914.81 (699.47) |
| Intrinsic motivation (T1) | 3.60 (0.77) | 3.61 (0.72) | - | - |
| Attitude cons (T2) | 3.93 (0.57) | 4.09 (0.54) | 3.82 (0.68) | 3.92 (0.75) |
| Attitude pros (T1) | 4.02 (0.53) | 4.00 (0.51) | - | - |
| Attitude pros (T2) | 3.88 (0.52) | 4.00 (0.55) | 3.89 (0.59) | 4.01 (0.51) |
| Self-efficacy (T2) | 3.71 (0.62) | 3.86 (0.72) | 3.85 (0.72) | 3.84 (0.71) |
| Action planning (T1) | 2.95 (1.02) | 2.95 (0.99) | - | - |
| Action planning (T2) | 2.92 (0.95) | 2.94 (0.97) | - | - |
| Coping planning (T2) | 2.58 (0.99) | 2.57 (0.99) | 2.49 (1.02) | 2.55 (1.00) |
| Strategic planning (T1) | 3.09 (0.59) | 3.10 (0.54) | - | - |
| Strategic planning (T2) | 3.00 (0.56) | 3.07 (0.58) | 3.01 (0.55) | 3.03 (0.52) |
| Strategic planning (T3) | 3.06 (0.55) | 3.12 (0.55) | - | - |
| Commitment (T1) | 3.69 (0.53) | 3.72 (0.51) | 3.66 (0.48) | 3.70 (0.53) |
| Commitment (T2) | 3.98 (0.61) | 4.03 (0.59) | 3.96 (0.60) | 4.01 (0.59) |
| Social support (T1) | 2.65 (1.26) | 2.82 (1.25) | - | - |
| Social support (T2) | 2.22 (0.88) | 2.50 (1.06) | 2.24 (0.98) | 2.70 (1.15) |
| Intention (T1) | - | - | 7.57 (1.49) | 7.58 (1.65) |
| Intention (T2) | 7.35 (1.65) | 7.72 (1.51) | 7.33 (1.76) | 7.59 (1.53) |
| Intention (T3) | 7.59 (1.62) | 7.79 (1.49) | 7.19 (1.89) | 7.49 (1.58) |
| Habit (T2) | 3.37 (0.78) | 3.53 (0.77) | 3.43 (0.88) | 3.55 (0.78) |
| Habit (T3) | 3.34 (0.83) | 3.54 (0.77) | 3.41 (0.92) | 3.58 (0.78) |
