# Supplementary material for: Bayesian Network Analysis of Intervention-Induced Physical Activity Behavior Change: Comparative Modeling Study Across Age, Education, and Activity Impairment Subgroups
Source: Online J Public Health Inform. 2025 Sep 3;17:e57977. doi: 10.2196/57977 (PMC12407225; doi:10.2196/57977)
Supplement: Multimedia Appendix 3 [file ojphi-v17-e57977-s003.docx]

| **Determinant (timeslot) / Subpopulation** | **Low education** | | **Medium to high education** | |
| --- | --- | --- | --- | --- |
|  | **Control** | **Intervention** | **Control** | **Intervention** |
| PA (T1) | 792.87 (732.10) | 901.30 (825.11) | 753.76 (703.88) | 847.66 (698.90) |
| PA (T2) | 819.90 (797.00) | 877.70 (767.63) | 727.61 (626.85) | 883.03 (770.07) |
| PA (T3) | 818.01 (840.09) | 867.68 (760.25) | 698.90 (658.79) | 787.32 (664.43) |
| Intrinsic motivation (T2) | - | - | 3.83 (0.64) | 3.89 (0.64) |
| Attitude cons (T1) | 3.90 (0.61) | 3.90 (0.65) | - | - |
| Attitude cons (T2) | 3.73 (0.67) | 3.87 (0.73) | 3.98 (0.60) | 4.06 (0.64) |
| Attitude pros (T1) | 3.93 (0.58) | 3.93 (0.57) | - | - |
| Attitude pros (T2) | 3.83 (0.61) | 3.97 (0.51) | 3.94 (0.51) | 4.03 (0.53) |
| Self-efficacy (T1) | 3.51 (0.74) | 3.63 (0.68) | - | - |
| Self-efficacy (T2) | 3.74 (0.76) | 3.78 (0.70) | 3.86 (0.62) | 3.89 (0.72) |
| Action planning (T1) | 2.77 (1.04) | 2.88 (1.02) | - | - |
| Action planning (T2) | - | - | 2.90 (0.95) | 2.93 (0.97) |
| Coping planning (T2) | - | - | 2.54 (0.98) | 2.52 (0.98) |
| Strategic planning (T1) | 3.05 (0.52) | 3.12 (0.51) | - | - |
| Strategic planning (T2) | 3.00 (0.52) | 3.07 (0.54) | 3.02 (0.59) | 3.04 (0.57) |
| Commitment (T1) | 3.61 (0.43) | 3.67 (0.47) | - | - |
| Commitment (T2) | 3.97 (0.60) | 4.07 (0.56) | 3.97 (0.62) | 4.00 (0.61) |
| Social modelling (T1) | 3.73 (0.92) | 3.71 (1.01) | - | - |
| Social modelling (T2) | 3.57 (1.00) | 3.57 (1.03) | - | - |
| Social support (T1) | 2.44 (1.20) | 2.61 (1.22) | - | - |
| Social Support (T2) | 2.29 (1.10) | 2.73 (1.19) | 2.19 (0.78) | 2.61 (1.08) |
| Intention (T1) | 7.55 (1.55) | 7.69 (1.54) | - | - |
| Intention (T2) | 7.17 (1.78) | 7.58 (1.57) | 7.45 (1.68) | 7.71 (1.50) |
| Intention (T3) | 7.24 (1.75) | 7.52 (1.59) | 7.47 (1.83) | 7.78 (1.50) |
| Habit (T2) | 3.44 (0.86) | 3.62 (0.71) | 3.35 (0.85) | 3.48 (0.81) |
| Habit (T3) | 3.44 (0.86) | 3.62 (0.76) | 3.30 (0.92) | 3.50 (0.79) |
