# Supplementary material for: Bayesian Network Analysis of Intervention-Induced Physical Activity Behavior Change: Comparative Modeling Study Across Age, Education, and Activity Impairment Subgroups
Source: Online J Public Health Inform. 2025 Sep 3;17:e57977. doi: 10.2196/57977 (PMC12407225; doi:10.2196/57977)
Supplement: Multimedia Appendix 4 [file ojphi-v17-e57977-s004.docx]

| **Determinant (timeslot) / Subpopulation** | **Without impairment** | | **With impairment** | |
| --- | --- | --- | --- | --- |
|  | **Control** | **Intervention** | **Control** | **Intervention** |
| PA (T1) | 809.70 (724.49) | 918.10 (736.85) | 714.61 (681.23) | 837.81 (817.50) |
| PA (T2) | 853.25 (718.22) | 947.10 (765.54) | 721.38 (744.84) | 854.23 (815.38) |
| PA (T3) | 824.75 (763.22) | 863.40 (744.82) | 699.23 (734.89) | 787.09 (690.38) |
| Intrinsic motivation (T1) | 3.64 (0.75) | 3.62 (0.72) | 3.52 (0.75) | 3.56 (0.72) |
| Attitude cons (T1) | 4.08 (0.55) | 4.08 (0.56) | 3.88 (0.60) | 3.84 (0.66) |
| Attitude cons (T2) | 3.84 (0.66) | 4.07 (0.64) | - | - |
| Attitude pros (T1) | - | - | 3.93 (0.55) | 3.84 (0.59) |
| Attitude pros (T2) | 3.89 (0.59) | 4.03 (0.53) | 3.87 (0.51) | 3.96 (0.51) |
| Self-efficacy (T1) | 3.71 (0.61) | 3.77 (0.64) | 3.44 (0.75) | 3.56 (0.72) |
| Self-efficacy (T2) | 3.82 (0.72) | 3.91 (0.72) | 3.77 (0.67) | 3.74 (0.69) |
| Action planning (T1) | 2.92 (1.03) | 2.91 (1.02) | 2.72 (1.04) | 2.78 (1.02) |
| Action planning (T2) | 2.90 (0.97) | 2.91 (0.96) | 2.67 (1.00) | 2.86 (1.00) |
| Coping planning (T1) | 2.57 (0.99) | 2.46 (0.96) | 2.62 (0.99) | 2.62 (0.97) |
| Coping planning (T2) | - | - | 2.54 (0.98) | 2.68 (0.97) |
| Strategic planning (T1) | 3.12 (0.56) | 3.09 (0.54) | 2.98 (0.56) | 3.06 (0.52) |
| Strategic planning (T2) | 3.02 (0.57) | 3.04 (0.56) | 2.98 (0.54) | 3.07 (0.54) |
| Strategic planning (T3) | 3.09 (0.58) | 3.12 (0.54) | - | - |
| Commitment (T1) | 3.64 (0.51) | 3.70 (0.50) | 3.74 (0.53) | 3.76 (0.55) |
| Commitment (T2) | 4.00 (0.60) | 4.03 (0.58) | 3.93 (0.61) | 4.00 (0.60) |
| Social modelling (T1) | - | - | 3.89 (0.88) | 3.83 (0.97) |
| Social support (T1) | 2.46 (1.20) | 2.68 (1.23) | - | - |
| Social support (T2) | 2.25 (0.98) | 2.68 (1.14) | - | - |
| Intention (T1) | 7.86 (1.55) | 7.95 (1.43) | 7.55 (1.52) | 7.53 (1.62) |
| Intention (T2) | 7.48 (1.65) | 7.73 (1.49) | 7.22 (1.77) | 7.56 (1.53) |
| Intention (T3) | 7.65 (1.65) | 7.84 (1.44) | 7.12 (1.84) | 7.44 (1.62) |
| Habit (T2) | 3.44 (0.79) | 3.56 (0.74) | - | - |
| Habit (T3) | 3.44 (0.83) | 3.61 (0.73) | - | - |
